# A more-Comers populAtion trEated with an ultrathin struts polimer-free Sirolimus stent: an Italian post-maRketing study (the CAESAR registry)

**DOI:** 10.3389/fcvm.2023.1326091

**Published:** 2024-01-08

**Authors:** Giuseppe Tarantini, Francesco Cardaioli, Giuseppe De Iaco, Bernardino Tuccillo, Maria Carmen De Angelis, Ciro Mauro, Marco Boccalatte, Antonio Trivisonno, Flavio Ribichini, Giuseppe Vadalà, Giuseppe Caramanno, Marco Caruso, Mario Lombardi, Dionigi Fischetti, Alessandro Danesi, Leonardo Abbracciavento, Giulia Lorenzoni, Dario Gregori, Andrea Panza, Luca Nai Fovino, Giovanni Esposito

**Affiliations:** ^1^ Department of Cardiac, Thoracic, Vascular Sciences and Public Health, University of Padua, Padova, Italy; ^2^ Department of Cardiology, Hospital “Cardinal G. Panico”, Tricase, Italy; ^3^ U.O.C. Cardiologia, P.O. Ospedale del Mare, Napoli, Italy; ^4^ Department of Cardiology, Hospital Cardarelli, Naples, Italy; ^5^ Interventional Cardiology Unit, Ospedale Santa Maria Delle Grazie Pozzuoli, Napoli, Italy; ^6^ Department of Cardiovascular Disease, “Antonio Cardarelli” Hospital, Campobasso, Italy; ^7^ Division of Cardiology, AOUI Verona, Verona, Italy; ^8^ Division of Cardiology, University Hospital Paolo Giaccone, Palermo, Italy; ^9^ Interventional Cardiology, San Giovanni di Dio Hospital, Agrigento, Italy; ^10^ Interventional Cardiology Unit, ARNAS Civico, G. Di Cristina Benfratelli, Palermo, Italy; ^11^ Interventional Cardiology Unit, A.O. Riuniti Villa Sofia-Cervello, Palermo, Italy; ^12^ Division of Cardiology, “V. Fazzi” Hospital, Lecce, Italy; ^13^ Division of Cardiology, S. Spirito Hospital, Rome, Italy; ^14^ Interventional Cardiology Unit, SS Annunziata Hospital, Taranto, Italy; ^15^ Unit of Biostatistics, Epidemiology and Public Health, University of Padova, Padova, Italy; ^16^ Division of Cardiology, Università Degli Studi di Napoli Federico II, Napoli, Italy

**Keywords:** coronary artery disease, PCI, ultrathin struts, acute coronary syndrome (ACS), drug-eluting stent (DES)

## Abstract

**Introduction:**

The use of contemporary drug-eluting stents (DES) has significantly improved outcomes of patients with coronary artery disease (CAD) undergoing percutaneous coronary intervention (PCI). However, concerns exist regarding the long-term proinflammatory effects of durable polymer coatings used in most DES, potentially leading to long-term adverse events. First-generation polymer-free stent technologies, such as sirolimus- and probucol-eluting stents (PF-SES), have shown an excellent safety and efficacy profile. The aim of this study was to evaluate the safety and efficacy of the new ultrathin Coroflex ISAR NEO PF-SES, in a more-comers PCI population.

**Methods:**

The CAESAR (a more-Comers populAtion trEated with an ultrathin struts polimer-free Sirolimus stent: An Italian post-maRketing study) registry is a multicenter, prospective study conducted in Italy, enrolling more-comers CAD patients undergoing PCI with the Coroflex ISAR NEO stent. Patients with left main (LM) disease, cardiogenic shock (CS), or severely reduced left-ventricular ejection fraction (LVEF) were excluded. The primary endpoint was target-lesion revascularization (TLR) at 1 year.

**Results:**

A total of 425 patients were enrolled at 13 centers (mean age 66.9 ± 11.6 years, Diabetes mellitus 29%, acute coronary syndrome 67%, chronic total occlusion 9%). Of these, 40.9% had multivessel disease (MVD) and in 3.3% cases, the target lesion was in-stent restenosis (ISR). Clinical device success was reached in 422 (99.6%) cases. At 1 year, only two (0.5%) subjects presented ischemia-driven TLR. The 1-year rates of target vessel revascularization and MACE were 0.5% and 5.1%, respectively. Major bleeding was observed in four (1.0%) patients.

**Conclusion:**

In this multicenter, prospective registry, the use of a new ultrathin Coroflex ISAR NEO PF-SES in a more-comers PCI population showed good safety and efficacy at 1 year.

## Introduction

The development of contemporary drug-eluting stents (DES) has considerably improved clinical outcomes compared with both bare-metal stents (BMS) and earlier DES platform iterations (1–3). As a result, international guidelines now advocate the use of last-generation DES for patients with coronary artery disease (CAD) necessitating percutaneous coronary intervention (PCI) (4). However, concerns exist regarding the potential long-term pro-inflammatory effects of durable polymer coatings typically employed for drug release regulation of most contemporary DES, possibly leading to neo-atherosclerotic phenomena (2, 5, 6).

To overcome these drawbacks, polymer-free stent technologies have been implemented. A polymer-free stent eluting sirolimus and probucol (PF-SES) has been shown to be non-inferior to a new-generation polymer-based zotarolimus-eluting stent at long-term follow-up (2). In addition, sirolimus and probucol have been assessed on an ultrathin bare-metal platform, revealing favorable safety and efficacy profiles in a more-comers population (7). The Coroflex ISAR NEO (B. Braun, Melsungen, Germany) is the latest released ultrathin PF-SES platform, introducing a restyled design aimed at improving trackability and maximal stent expansion.

The aim of this study was to assess the safety and efficacy of the Coroflex ISAR NEO platform in a more-comers population including patients with *de novo* and restenotic lesions, both in native coronary arteries and coronary bypass grafts (CABG).

## Methods

### Study design and patient population

The CAESAR (a more-Comers populAtion trEated with an ultrathin strut polymer-free Sirolimus stent: An Italian post-maRketing study) registry is a prospective, national, multicenter, postmarket study enrolling patients with CAD undergoing PCI with the Coroflex ISAR NEO coronary stent at 13 Italian centers. According to the study protocol, we included patients ≥18 years of age with an acute coronary syndrome (ACS), chronic coronary syndrome (CCS), or objective proof of ischemia meeting the requirements for PCI (4). Either single- or multivessel stenting was allowed for treating *de novo* or restenotic lesions (reference diameters from 2.0 to 4.0 mm), both in native coronary arteries and CABG. Limited exclusion criteria were considered for the study [left main (LM) disease, cardiogenic shock (CS), severe calcified stenoses, and severely reduced left-ventricular ejection fraction (LVEF)], resulting in a more comprehensive cohort of PCI patients (more-comers). The complete study inclusion and exclusion criteria are provided in Table 1.

**Table 1:** Inclusion and exclusion criteria.

Inclusion criteria
– *De novo* and restenotic lesions in native coronary arteries and coronary bypass grafts with reference vessel diameter ≥2 and ≤4 mm, with suitable lesion length (target lesion lengths >34 mm need to be covered with at least 2 stents) – Subjects ≥18 years of age
Exclusion criteria
– Intolerance to sirolimus and/or probucol – Allergy to components of the coating – Pregnancy and lactation – Severely calcified stenosis – Cardiogenic shock – Hemorrhagic diathesis or another disorder such as gastrointestinal ulceration or cerebral circulatory disorders, which restrict the use of antiplatelet or anticoagulation therapy – Planned surgery ≤6 months after myocardial revascularization – Severe allergy to contrast media – Patients with an ejection fraction of <30% – Reference vessel diameter <2 and >4 mm – Treatment of the left main stem – Indication for bypass surgery – Contraindication for whichever accompanying medication is necessary

### Study device and procedure

The Coroflex ISAR NEO is the latest-generation PF-SES. Similarly to its predecessor (Coroflex ISAR), it is built on a premounted cobalt-chrome (Co-Cr) alloy ultrathin platform (7), while it presents a redesigned strut structure that enables greater stent expansion, maintaining at the same time good flexibility and radial force. The Coroflex ISAR NEO is available in 2.0 mm (55 µm thick) and 3.5 mm (65 µm thick) diameter sizes with a maximal expansion capability of up to 5.0 mm. Lesion pre-dilation was left to the discretion of the operators. The antiplatelet therapy after PCI had to follow the latest guidelines (4, 8–10). A dedicated electronic data capture system was used for data entry, and data accuracy was verified by the principal investigators at each center. Device success is defined as the successful delivery and deployment of the device and attainment of <50% diameter stenosis using only the study device. Procedural success was defined as a successful stent deployment with an antegrade thrombolysis in myocardial infarction (TIMI) grade 3 flow at the end of the procedure.

### Study endpoints

The primary endpoint (EP) was target lesion revascularization (TLR) at 1 year defined as ischemia-driven revascularization of the target lesion (within the stent or the 5 mm borders adjacent to the stent). Secondary EPs were all-cause mortality, definite stent thrombosis (ST), bleeding events, major adverse cardiovascular events [MACE, a composite of all-cause and cardiac death, myocardial infarction (MI), and TLR], and target vessel failure [TVF, a composite of cardiac death, MI attributed to the target vessel, or target vessel revascularization (TVR)]. Myocardial infarctions were defined according to the fourth universal MI definition (11). The follow-up was prospectively performed at 30 days (±7 days) and 1 year (±30 days) with outpatient visits or telephone interviews. The definitions of individual endpoints can be found in the study protocol (Supplementary Appendix).

### Statistical analysis

For the CAESAR study, the sample size calculation was based on the primary EP. The detailed description of the procedure regarding sample size calculation is reported in the Supplementary Material. Continuous variables are expressed as median [interquartile range (IQR)] and compared using the Mann–Whitney *U* test. Categorical variables are presented as counts (%) and were compared using the Chi-square test or Fisher's exact test. The Kaplan–Meier method was used to estimate survival and MACE-free survival at follow-up. Cumulative incidence functions (CIFs) were used to evaluate bleeding at follow-up accounting for competing risks. For all the analyses, a two-sided *p* < 0.05 was considered to be significant. The analyses were performed with the R software (R Foundation, Wien, Austria).

## Results

### Baseline clinical characteristics

Between August 2019 and March 2021, a total of 425 patients were enrolled in the CAESAR study at 13 centers. Detailed demographics and clinical characteristics are described in Table 2. In summary, patients were aged 66.9 ± 11.6 years and 75% were male. Diabetes mellitus was present in 29% of the patients (11% insulin-dependent). One-third of the study population had undergone previous percutaneous coronary artery revascularization. The median baseline LVEF was 55%. Clinical indications for PCI were ACS in 67% of the patients (>50% of the subjects were admitted because of acute MI), and 3% of them had a New York Heart Association (NYHA) Functional Class III–IV on admission.

**Table 2:** Baseline clinical characteristics.

Clinical characteristics	Overall population
	(*n* = 425)
Age (years)	66.96 (11.16)
Female gender	108 (25.4)
BMI (kg/m^2^)	27.31 (24.9–29.4)
Hypercholesterolemia	382 (89.8)
Hypertension	351 (82.6)
Diabetes mellitus	124 (29.2)
IDDM	49 (39.5)
Dialysis	5 (1.2)
Smoking	
Current	118 (27.8)
Former	118 (27.8)
Previous MI	105 (24.7)
Atrial fibrillation	52 (12.1)
Previous coronary revasc.	125 (29.4)
LVEF	55 (45–60)
Previous stroke/TIA	12 (2.8)
COPD	30 (7.1)
Peripheral artery disease	55 (13.0)
Stress test	45 (10.6)
Clinical presentation	
Unstable angina	62 (14.6)
NSTEMI	109 (25.5)
STEMI	121 (28.5)
CCS	106 (25.0)
Other	27 (6.4)
NYHA class > 2	14 (3.3)
Angina	185 (43.5)
Angina class (CSS)	
1	40 (21.6)
2	90 (48.7)
3	55 (29.7)

Values are *n* (%), or median (range), as appropriate for categorical and continuous variables, respectively.

IDDM, insulin-dependent diabetes mellitus; COPD, chronic obstructive pulmonary disease; CAD, coronary artery disease; LVEF, left-ventricular ejection fraction; MI, myocardial infarction; PCI, percutaneous coronary intervention; TIA, transient ischemic attack; NSTEMI, non-ST-elevation myocardial infarction; STEMI, ST-elevation myocardial infarction; CCS, chronic coronary syndrome.

### Angiographic and procedural characteristics

Overall, 492 lesions were treated with 484 PF-SES. Angiographic and procedural characteristics are reported in Tables 3, 4. A total of 492 lesions were treated in 425 patients, with 174 (40.9%) patients presenting multivessel disease (MVD). Left anterior descending (LAD) was involved in 43% of the cases. In 16 (3.4%) cases, the target lesion resulted from in-stent restenosis (ISR), while three lesions were located on a previous CABG. Chronic total occlusion (CTO) represented 9% of the total treated lesions. Coronary stenting was performed through a radial approach in 92.9% of the cases, mostly by using a 6F guiding catheter (98.8%). Pre- and post-dilation were performed in 76% and 66% of the cases, respectively. Clinical device success was reached in 422 (99.6%) patients. In 10 cases, the use of an adjunctive different stent platform was considered necessary. No intraprocedural strokes or deaths were reported. Furthermore, there were no recorded cases of acute bleeding or thrombotic complications. Four (1%) periprocedural MI were reported. A P2Y12 pretreatment strategy was applied in 235 (55.4%) patients. In 10 (2.4%) cases, intraprocedural Cangrelor infusion was administered. The time prescription for dual antiplatelet therapy (DAPT) at discharge was 1–3 months for 55 (13%) patients (all with an indication for OAC), 6 months for 75 (17.6%) subjects, and ≥12 months for the rest of the study population (Supplementary Table S1**)**. At 1-year follow-up, 53% of the patients were taking two antiplatelet drugs.

**Table 3:** Angiographic characteristics.

Angiographic characteristics	Total no. of patients
	(*n* = 425)
Multivessel disease	174 (40.9)
Total no. of lesions	(*n* = 492)
Lesion location	
LAD	215 (43.7)
LCX	143 (29.0)
RCA	130 (26.4)
Grafts	4 (0.9)
Bifurcation	53 (10.8)
Calcification	112 (22.8)
Intracoronary thrombus	100 (20.3)
Lesion type	
A	63 (12.8)
B1	179 (36.4)
B2	150 (30.5)
C	100 (20.3)
Total occlusion	44 (8.9)
Restenosis	16 (3.4)
Ostial target	27 (5.5)
SVG involvement	3 (0.6)
Severe tortuosity	20 (4.1)

Values are *n* (%), or median (range), as appropriate for categorical and continuous variables, respectively.

LM, left main coronary artery; LAD, left anterior descending coronary artery; LCX, left circumflex coronary artery; RCA, right coronary artery; SVG, saphenous vein graft.

**Table 4:** Procedural characteristics.

Procedural characteristics	Overall population
	(*n* = 425)
Vascular access	
Femoral	30 (7.1)
Radial	395 (92.9)
Guiding catheter	
6F	420 (98.8)
7F	5 (1.2)
N° of Coroflex ISAR/lesion	1.18 (0.32)
Lesion length (mm)	18.0 (9.0, 38.00)
Predilatation performed	323 (76.0)
Other DES	10 (2.4)
DEB	4 (0.9)
Two stent technique in bifurcation	9 (2.1)
Residual stenosis	
<40 (%)	402 (94.6)
41–69 (%)	21 (4.9)
>70%	2 (0.5)
Postdilatation performed	280 (66.0)
Final TIMI	
1	1 (0.2)
2	5 (1.2)
3	419 (98.6)
Clinical device success	422 (99.3)
Procedural success	425 (100.0)
Coronary perforation	0 (0.0)
Coronary dissection	4 (0.9)
Intraprocedural stroke	0 (0.0)
Intraprocedural death	0 (0.0)
Periporocedural MI	4 (1)
Use of IABP, ECMO, LVAD	
Planned	2 (0.5)
Unplanned	2 (0.5)
Bleeding complications	0 (0.0)
Acute intrastent thrombosis	0 (0.0)
Previous ASA treatment	337 (79.3)
P2Y12 pretreatment	235 (55.4)
Clopidogrel	72 (30.7)
Prasugrel	4 (1.7)
Ticagrelor	159 (67.6)
Chronic OAC treatment	94 (22.1)
GP IIb/IIIa inhibitor	37 (8.7)
Cangrelor	10 (2.4)

Values are *n* (%), or median (range), as appropriate for categorical and continuous variables, respectively.

DES, drug-eluting stent; DEB, drug-eluting balloon; MI, myocardial Infarction; IABP, intra-aortic balloon pump; ECMO, extra-corporeal membraneous oxygenation; LVAD, left ventricular assistance device; ASA, acetylsalicylic acid; PCI, percutaneous coronary intervention; OAC, oral anticoagulant.

### Outcomes

**Figure 1:**
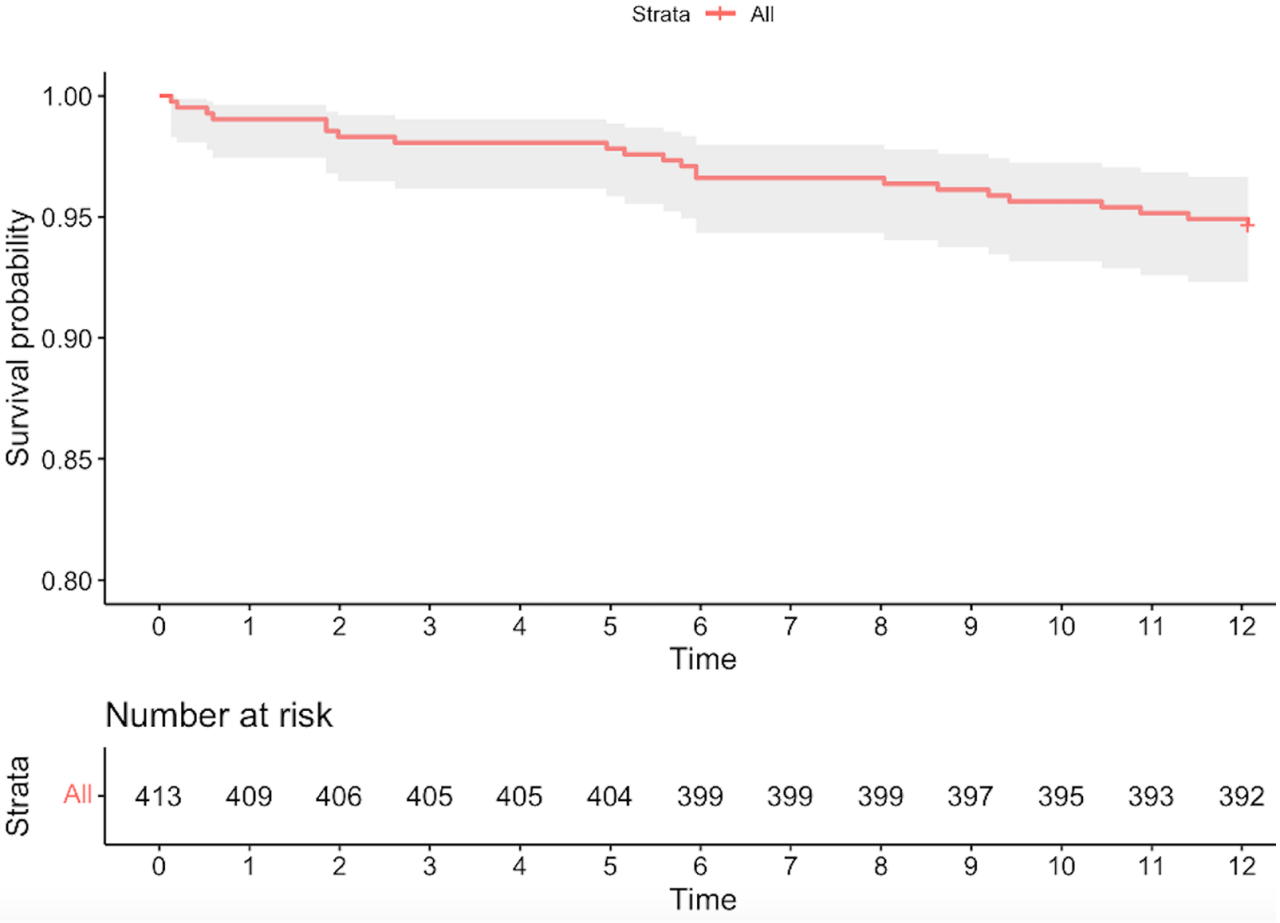
The Kaplan–Meier curve for MACE.

The rates of primary and secondary EP at different time points are provided in Table 5 and Supplementary Tables S2, S3. The 1-year follow-up was available for 410 (96.4%) patients. Only two (0.5%) subjects presented the primary EP, of those one was an early MI related to the target lesion, treated with PCI. Out of the 20 deaths (4.9%), one (5%) was cardiac in nature. The Kaplan–Meier curve for all-cause death is reported in Supplementary Figure S1. The 1-year rates of TVR and MACE were 0.5% (CI 0.1–0.9) and 6.1% (CI 4.6–7.2), respectively (Figure 1), while no ST was reported. Major bleeding was observed in four (1.0%) patients (Supplementary Figure S2).

**Table 5:** One-year follow-up outcomes.

One-year follow-up outcomes	Overall population
	(*n* = 410)
TLR	2 (0.5)
PCI	2 (100.0)
Cumulative MACE	26 (6.1)
Death	20 (4.9)
Cardiac	1 (5.0)
Non-cardiac	19 (95.0)
TVR	2 (0.5)
PCI	2 (100.0)
MI	5 (1.1)
TVF	3 (0.7)
Stent thrombosis	0 (0.0)
Angina	8 (1.9)
Angina class (CSS)	
1	2 (25.0)
2	3 (37.5)
3	3 (37.5)
Stroke	1 (0.2)
Bleeding	4 (1.0)
BARC 2	3 (75.0)
BARC 3b	1 (25.0)

Values are *n* (%), or median (range), as appropriate for categorical and continuous variables, respectively.

ASA, acetylsalicylic acid; DAPT, dual antiplatelet therapy; MI, myocardial infarction; TLR, target lesion revascularization; TVR, target vessel revascularization; TVF, target vessel failure.

## Discussion

The CAESAR study was the first real-world registry to evaluate the performance of the new polymer-free, ultrathin, sirolimus- and probucol-eluting stent (Coroflex ISAR NEO) in a more-comers population.

The key findings of this study can be summarized as follows: (1) The Coroflex ISAR NEO stent demonstrated excellent procedural performance, with high rates of procedural and clinical success, (2) At 1-year follow-up, TLR, TVR, and ST rates were very low.

The advent of DES marked a pivotal step in preventing in-stent restenosis. However, early-generation DES demonstrated an increased risk of both late and very late stent thrombosis when compared with BMS (12, 13). This was likely attributable to compromised arterial healing subsequent to stent implantation, a consequence of inflammatory reaction associated with the polymer and endothelial cell dysfunction (14–16). In response, polymer-free (PF) stents were introduced to the market. The initial generations of PF stents displayed lower clinical effectiveness in comparison with the durable polymer DES, potentially owing to overly rapid drug elution without polymer control (17, 18). This challenge was effectively addressed in recent years by incorporating a secondary molecule to regulate drug release.

One such approach is the probucol- and sirolimus-coated polymer-free stents (19, 20). The first iteration exhibited favorable long-term (10-year) safety and efficacy when compared with high-performance durable polymer DES (2). The second generation of PF-SES (Coroflex ISAR), characterized by ultrathin struts (50/60 µm), was successfully tested in the international ISAR 2000 registry, demonstrating low event rates at 9-month follow-up in both CCS and ACS patients (7). Moreover, thanks to their ultrathin struts and the pro-reendothelializing effects of probucol, this type of device exhibited improved strut coverage at 3 months compared with a last-generation sirolimus-eluting stent with a bioresorbable degradable polymer (21).

The CAESAR registry was the first to evaluate the latest generation of PF-SES, the Coroflex ISAR NEO. In comparison with its predecessor, the new stent features a distinct structural backbone design that enhances visibility during the procedure and allows for broader expansion while maintaining radial stability, flexibility, strut thickness, and coating characteristics.

The device's procedural success reported in the present study was excellent, even in the presence of a high degree of target lesion complexity (>50% of patients presenting with type B2/C lesions, and almost 10% of treated lesions were CTOs), suggesting favorable stent performance in challenging anatomies as well. Regarding clinical outcomes, very low rates of adverse events were recorded during follow-up. Specifically, only two patients experienced the primary endpoint (TLR), and no case of stent thrombosis was reported. Comparing these findings with previous registries and trials involving PF-SES (as well as other platforms with ultrathin struts), the adverse stent-related event rate in this registry was notably lower (2, 7, 19, 22). This could be attributed to several factors, potentially synergistic: (1) the improved performance of the current device compared with its predecessors, (2) a relatively high 1-year mortality rate (especially due to non-cardiac causes), with death acting as a competing risk, and (3) the relatively small length of the treated lesions.

Nevertheless, the low rate of adverse events herein reported is reassuring in terms of the device's safety and efficacy profile. Furthermore, given the baseline high ischemic risk of the enrolled population (>50% of clinical presentations were ACS, and almost half of the enrolled patients had MVD), the limited number of ischemic events during follow-up is even more remarkable. The absence of mandatory angiographic follow-up might also explain the lower rates of TLR of the CAESAR study as compared with the previous trials [such as the ISAR-TEST 5 (7, 19)], potentially reducing the number of PCI indicated at angiographic follow-up.

Furthermore, it should be noted that, despite the baseline population's high ischemic risk (almost 70% of the cases were admitted because of unstable CAD), only 53% of the patients included in the study were prescribed a DAPT regimen at 12 months. Accordingly, the 1-year rate of major bleeding events was low and comparable with other previous registries (2, 7, 19, 22). Although the observational nature of the present analysis, along with its small sample size, does not allow for robust subanalysis on the impact of different DAPT regimens, the low event rates observed are encouraging.

The reported data must be viewed in light of the numerous inherent limitations of the study. Primarily, the potential sample size underestimation, combined with the less stringent data monitoring inherent to an observational study, might represent an adverse event under-reporting bias. As a consequence, the present results did not permit the execution of any subgroup analysis of those prespecified according to the study protocol. Secondly, the absence of a core laboratory for standardized assessment of baseline and procedural angiographies did not allow for a standardized angiogram evaluation. Similarly, the absence of an independent adjudication committee could have led to under-reported adverse event rates. Thirdly, the absence of angiographic follow-up and intravascular imaging data could have led to a reduced number of TLR reports and prevented a detailed struts coverage evaluation. Yet, indications of PCI for ISR in asymptomatic patients remain debatable. Furthermore, considering the relatively short follow-up period, very late stent thrombosis is not included as an endpoint in the current study and will be evaluated in subsequent analyses with a longer follow-up. Finally, it is important to note that the use of different DES platforms was necessary in 10 cases, potentially leading to biased outcomes. In conclusion, due to the specific inclusion and exclusion criteria and the utilization of a predetermined study platform, the findings from this analysis should not be generalized to more complex populations (e.g., severely calcified lesions or challenging anatomy) or to other devices, whether ultrathin or PF stents, which were not considered in this study.

## Conclusions

The prospective CAESAR registry demonstrated the optimal safety and efficacy profile of the new-generation PF-SES Coroflex ISAR NEO in a more-comers population. This study contributes to the growing body of evidence supporting the use of PF-SES and showed promising results in terms of procedural success and low adverse event rates at 1-year follow-up. Further research is needed to confirm our results and explore the broader applicability of PF-SES in specific patient subsets, over an extended follow-up period.

## Data Availability

The original contributions presented in the study are included in the article and **Supplementary Material**. Further inquiries can be directed to the corresponding author.
