# Supplementary material for: A more-Comers populAtion trEated with an ultrathin struts polimer-free Sirolimus stent: an Italian post-maRketing study (the CAESAR registry)
Source: Front Cardiovasc Med. 2024 Jan 8;10:1326091. doi: 10.3389/fcvm.2023.1326091 (PMC10828965; doi:10.3389/fcvm.2023.1326091)
Supplement: Supplementary file 1 [file Table1.docx]

**Supplementary Table 1.** Anti-thrombotic therapy (Discharge, 30-days, 1 year).

| **Discharge** | **Overall population**  (n = 425) |
| --- | --- |
| ASA | 413 (97.2) |
| P2Y12 inhibitors | 419 (98.6) |
| Clopidogrel | 171 (40.8) |
| Prasugrel | 24 (5.7) |
| Ticagrelor | 222 (53.0) |
| Ticlopidine | 2 (0.5) |
| Oral anticoagulation | 55 (13.0) |
| DAPT intended time (months) |  |
| 1-3 | 55 (13.0) |
| 6 | 75 (17.6) |
| 12 | 295 (69.4) |
| **30 days** | **Overall population**  (n = 425) |
| ASA | 403 (94.8) |
| P2Y12 inhibitors | 405 (95.3) |
| Clopidogrel | 167 (41.2) |
| Prasugrel | 24 (5.9) |
| Ticagrelor | 214 (52.9) |
| Ticlopidine | 0 (0.0) |
| Oral anticoagulation | 51 (12.0) |
| Reducing DAPT | 7 (1.6) |
| **1 year** | **Overall population**  (n = 410) |
| ASA | 339 (82.7) |
| P2Y12 inhibitors | 244 (59.5) |
| Clopidogrel | 108 (44.4) |
| Prasugrel | 7 (2.7) |
| Ticagrelor | 129 (52.9) |
| Ticlopidine | 0 (0.0) |
| Oral anticoagulation | 48 (11.7) |

*Values are n (%), or median (range), as appropriate for categorical and continuous variables, respectively.

*ASA: acetyl-salicilic acid; DAPT: dual antiplatelet therapy*

**Supplementary Table 2.** In-hospital outcomes.

| **In-hospital** | **Overall population**  (n = 425) |
| --- | --- |
| TLR | 1 (0.2) |
| MACE | 10 (2.3) |
| Death | 4 (0.9) |
| TVR | 1 (0.2) |
| MI after index procedure | 1 (0.2) |
| Periprocedural MI | 4 (0.9) |
| Stent thrombosis | 0 (0.0) |
| Stroke | 1 (0.2) |
| Bleeding | 1 (0.2) |

*Values are n (%), or median (range), as appropriate for categorical and continuous variables, respectively.

*MI: myocardial infarction; TLR: target lesion revascularization; TVR: target vessel revascularization; MACE: major adverse cardioacscular event*

**Supplementary Table 3.** 30-day follow up outcomes.

| **30-days follow-up outcomes** | **Overall population**  (n = 425) |
| --- | --- |
| TLR | 1 (0.2) |
| MACE | 11 (2.6) |
| Death | 5 (1.2) |
| TVR | 1 (0.2) |
| MI (_exluding periprocedural)_ | 1 (0.2) |
| Stent thrombosis | 0 (0.0) |
| Stroke | 1 (0.2) |
| Bleeding | 1 (0.2) |

*Values are n (%), or median (range), as appropriate for categorical and continuous variables, respectively.

*ASA: acetyl-salicilic acid; DAPT: dual antiplatelet therapy; MI: myocardial infarction; TLR: target lesion revascularization; TVR: target vessel revascularization.*
